# Non-Inflammasome Forming NLRs in Inflammation and Tumorigenesis

**DOI:** 10.3389/fimmu.2014.00169

**Published:** 2014-04-22

**Authors:** Irving Coy Allen

**Affiliations:** ^1^Department of Biomedical Sciences and Pathobiology, Virginia-Maryland Regional College of Veterinary Medicine, Virginia Polytechnic Institute and State University, Blacksburg, VA, USA

**Keywords:** Nod-like receptors, NLRP12, NLRX1, NLRC3, NF-κB, TRAF, cancer, pattern recognition receptors

## Abstract

Aberrant inflammation is an enabling characteristic of tumorigenesis. Thus, signaling cascades that alter inflammatory activation and resolution are of specific relevance to disease pathogenesis. Pattern recognition receptors (PRRs) are essential mediators of the host immune response and have emerged as critical elements affecting multiple facets of tumor pathobiology. The nucleotide-binding domain and leucine-rich repeat containing (NLR) proteins are intracellular PRRs that sense microbial and non-microbial products. Members of the NLR family can be divided into functional sub-groups based on their ability to either positively or negatively regulate the host immune response. Recent studies have identified a novel sub-group of non-inflammasome forming NLRs that negatively regulate diverse biological pathways associated with both inflammation and tumorigenesis. Understanding the mechanisms underlying the function of these unique NLRs will assist in the rationale design of future therapeutic strategies targeting a wide spectrum of inflammatory diseases and cancer. Here, we will discuss recent findings associated with this novel NLR sub-group and mechanisms by which these PRRs may function to alter cancer pathogenesis.

## Introduction

The intimate association between inflammation and cancer was first noted over 150 years ago by Rudolf Vierchow (1, 2). Indeed today, aberrant inflammation is considered both an emerging hallmark of tumorigenesis and an enabling characteristic of cancer (3). Tumorigenesis is a multistep process and inflammation functions at multiple levels to both antagonize and enhance tumor initiation and progression (3). During the early stages of tumorigenesis, an inflammatory microenvironment serves as an enabling characteristic to activate diverse signaling pathways and drive the progression of pre-malignant and malignant lesions toward cancer (3–5). In later stages, cancer cells typically acquire a diverse repertoire of defense mechanisms that allow the cells to both passively and actively evade immune surveillance and elimination (3, 6, 7). This immune system subversion is an emerging hallmark of cancer and serves to remove the most effective barriers employed by the host to defend against neoplasia, late-stage tumor, and micro-metastasis progression (3).

Pattern recognition receptors (PRRs) are an essential component of the host immune system and significantly contribute to cancer pathobiology. There are 4 major families of PRRs that have been implicated in tumorigenesis, including the toll-like receptors (TLRs), the nucleotide-binding domain and leucine-rich repeat containing (NLR) family of sensors, C-type lectin receptors (CLRs), and RIG-I-like receptors (RLRs) (8). These receptor families function to initiate inflammatory signaling cascades following the direct or indirect recognition of pathogens, damage and stress through sensing highly conserved pathogen-associated molecular patterns (PAMPs), and damage-associated molecular patterns (DAMPs). In addition to their roles in facilitating the immune response, PRRs also play fundamental roles in the regulation of proliferation, cell survival and death, reactive oxygen species generation, angiogenesis, and tissue remodeling and repair (8). In the context of cancer, PRRs drive the immune response following exposure to potentially carcinogenic pathogens, environmental exposures to mutagenic agents and insults, and cancer-associated cellular damage and stress (9–16). In general, increased PRR signaling creates an enriched, pro-inflammatory microenvironment that is favorable for tumor initiation and progression (17). Thus, we find that PRRs are stuck in a “Goldilocks Conundrum.” Robust PRR activation is critical in driving the host immune response following PAMP and DAMP exposure; whereas, an overzealous and persistent immune response driven by PRR activation can cause significant collateral damage to the host tissue that ultimately results in chronic inflammation and cancer.

To date, the majority of studies evaluating PRR signaling in cancer have focused on members of the TLR family. However, new and emerging findings have revealed a significant role for members of the NLR family in contributing either directly or indirectly to a variety of hallmarks associated with cancer, including inflammation, cell death, tumor growth, angiogenesis, invasion, and metastasis (18–26). There are at least 23 distinct NLR and NLR-like proteins that have been identified in humans and 34 family members identified in mice (23, 27–29). The NLR proteins function as cytosolic receptors and sensors to detect intracellular PAMPs and DAMPs. Since their discovery, a variety of names have been used to describe the members of this gene family and their respective proteins. For example, these PRRs have been previously referred to as CATERPILLERs, NOD-like receptors, NACHT-leucine-rich repeats (LRR), and NBD-LRR proteins (28). This resulted in a lack of consistency in the field and resulted in the currently accepted and standardized nomenclature defining the NLRs as the NLR gene family (28). These proteins contain a highly conserved tripartite domain structure (28). The N-terminal domain of the protein is comprised of a variable, but limited number of effector domains that can include combinations of acidic transactivation domains (NLRA proteins), baculoviral inhibitory repeat (BIR)-like domains (NLRB proteins), caspase recruitment domains (NLRC proteins), and pyrin domains (NLRP proteins) (28). These N-terminal domains function to recruit adaptor, intermediary, or effector molecules that drive downstream signaling. The core of the protein is comprised of a conserved NACHT nucleotide-binding domain, which facilitates oligomerization (28). The C-terminal domain of the protein contains multiple LRR elements, which are essential for ligand sensing (28). Each LRR element is typically 28–29 residues in length and each NLR may contain up to 33 individual LRR elements (30, 31).

## Inflammasome Forming NLRs in Cancer

One of the most fundamental roles of the NLR family is to regulate pro-inflammatory cytokines and chemokines that drive the host innate immune response to pathogens and environmental insults. Key to this response is the proper regulation of IL-1β and IL-18, which are both potent pro-inflammatory cytokines that affect diverse aspects of health and disease (32–37). Both of these cytokines are generated in an immature pro-form that requires post-translational cleavage for activation. A functional sub-group of NLRs has been identified as driving this process through the formation of a multi-protein complex termed the inflammasome (32, 35, 36). Upon activation, the NLR is thought to undergo a conformational change that allows the recruitment and binding of adaptor and effector proteins and inflammasome formation (35). The inflammasome is composed of an NLR that recognizes a specific repertoire of PAMPs and DAMPs, the adaptor protein ASC, and pro-Caspase-1 (32). These sub-units continue to multiplex, ultimately resulting in the maturation and activation of Caspase-1, which subsequently drives the cleavage and activation of IL-1β and IL-18. These inflammasome forming NLRs are by far the best characterized and most highly studied members of the NLR family. To date, at least 6 NLR and NLR-like proteins have been strongly implicated in inflammasome formation, including NLRP1, NLRP3, NLRP6, NLRC4, NLRC5, and the PYHIN family member AIM2 (NLR-like) (32–37). Inflammasome forming NLRs significantly regulate the tumor microenvironment by modulating cytokine production. For example, many of the inflammasome forming NLRs have been shown to significantly attenuate inflammation and tumorigenesis in mouse models of colitis-associated colorectal cancer (CAC) by regulating IL-18 production (18, 19, 21, 22, 38–40). In addition to being a potent pro-inflammatory cytokine, IL-18 is also secreted by epithelial cells to stimulate regeneration and repair and improve barrier function in the colon, thus loss of this cytokine in NLR inflammasome deficient mice enhances tumorigenesis (41). Beyond colon cancer, NLR inflammasome activation may also play important roles in many other types of cancer, including breast cancer, skin cancers, and virus-associated hepatocellular carcinoma (25, 26, 42–47).

## Non-Inflammasome Forming NLRs That Negatively Regulate Inflammation

**Figure 1:**
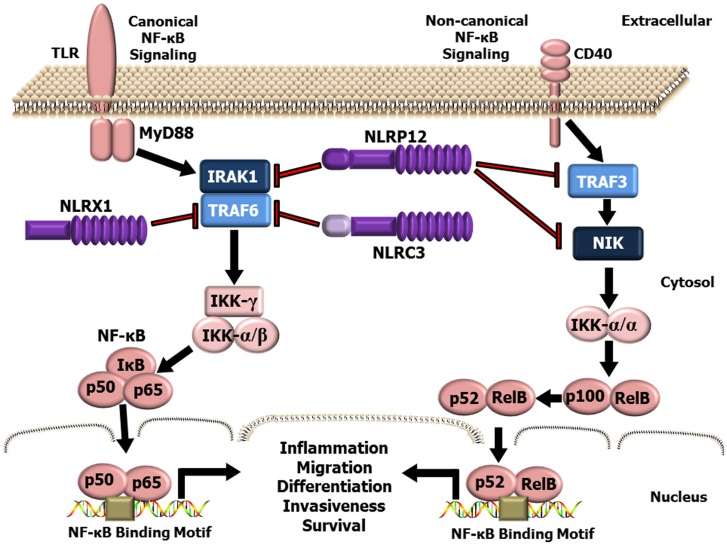
**Schematic illustrating NLR attenuation of canonical and non-canonical NF-κB signaling**. NF-κB is a master regulator of gene transcription and contributes to several hallmarks of cancer. NLRX1, NLRP12, and NLRC3 negatively regulate NF-κB signaling at multiple levels. NLRX1 interacts with and inhibits TRAF6 and the IKK complex resulting in the attenuation of NF-κB signaling following TLR stimulation. Likewise, NLRC3 was also shown to interact with TRAF6 and attenuate NF-κB signaling through a similar mechanism. NLRP12, has been shown to attenuate both the canonical NF-κB signaling pathway through modulating the phosphorylation of IRAK-1 and the non-canonical NF-κB pathway through interactions with TRAF3 and NIK.

While the inflammasome forming NLRs are the best characterized members of this PRR family, recent studies have identified a functional sub-group of NLRs that negatively regulate inflammation (48–54). This sub-group is currently composed of three NLR family members, NLRP12, NLRX1, and NLRC3 (Figure 1). NLRP12 was one of the first NLR proteins to be described and is the best characterized member of this functional NLR sub-group. NLRP12 was previously known as monarch-1 and PYPAF7 and was originally suggested to form an inflammasome with ASC in overexpression systems (55, 56). In these overexpression studies, transient transfection of NLRP12 and ASC was also shown to induce the transcription of an NF-κB reporter construct (56). Thus, these early *in vitro* studies initially suggested that NLRP12 was an inflammasome forming NLR and a positive regulator of NF-κB signaling. These findings are also consistent with human data that has identified mutations in NLRP12 linked to a spectrum of hereditary periodic fever syndromes. The disorders associated with *NLRP12* mutations are characterized by redox alterations and enhanced secretion of IL-1β, which are similar to the characteristics associated with the family of diseases linked to gain-of-function mutations in the *NLRP3* gene (57–59). Interestingly, these diseases are associated with increased caspase-1 activity, are sensitive to therapeutics targeting IL-1β (anakinra), and appear to be independent of NF-κB activation (57–59). However, the ability of NLRP12 to form a functional inflammasome under physiological situations and in the context of human disease appears to occur only under highly specific conditions and is an area of current investigation (60, 61). Indeed, several studies have evaluated NLRP12 inflammasome formation *ex vivo* and using *Nlrp12*^−/−^ mice under a variety of conditions and have directly shown that this NLR does not regulate IL-1β/IL-18 maturation (62–69). The prevailing literature associated with NLRP12 indicates that this protein functions as a negative regulator of inflammation by modulating canonical and non-canonical NF-κB signaling (48, 49, 62, 65, 66, 68, 70–73). NLRP12 negatively regulates non-canonical NF-κB signaling through its association with TRAF3 and NF-κB inducing kinase (NIK) (49, 68). This interaction leads to the degradation of NIK and subsequent attenuation of p100 cleavage to p52 (Figure 1). Similarly, NLRP12 attenuates canonical NF-κB signaling through the inhibition of IRAK-1 phosphorylation (48, 66, 71) (Figure 1). In addition to directly mediating the NF-κB cascade, NLRP12 has also been shown to attenuate ERK signaling, though the exact mechanism has yet to be fully resolved (66, 68). Thus, while some conflicting data has been reported, most issues can be resolved by considering the technical limitations of the assays used to define the respective mechanisms and the specific models being evaluated.

**Figure 2:**
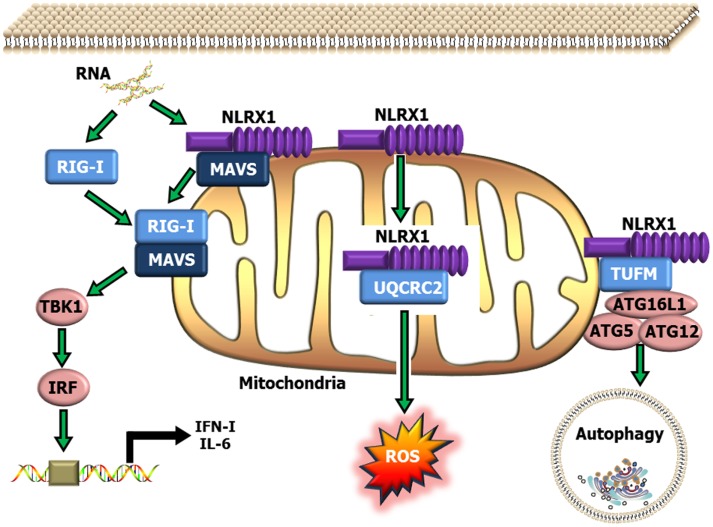
**Schematic illustrating NLRX1 regulation of type-I interferon, ROS and autophagy signaling**. NLRX1 is localized to the mitochondria, where it has been shown to bind with MAVS and prevent the interaction between MAVS and RIG-I during the host anti-viral response. This interaction significantly attenuates MAVS activation of IRF3 and IRF7 and results in reduced IFN and IL-6 signaling. NLRX1 has also been shown to function as a positive regulator of autophagy through its interactions with the mitochondrial protein TUFM, and the mitochondrial immune signaling complex (MISC), which includes Atg5–Atg12 and ATG16L1. This complex has been shown to be important in promoting virus-induced autophagy and concurrently attenuating IFN signaling. In addition to its role in attenuating host anti-viral signaling, NLRX1 has also been shown to significantly augment ROS generation from the mitochondria through interactions with UQCRC2 following infection with specific species of bacteria.

NLRX1 was originally characterized in 2008, and was shown to negatively regulate the host anti-viral immune response (51). NLRX1 is unique among the NLRs due to its mitochondrial localization and its relatively undefined N-terminal domain. Similar to NLRP12, NLRX1 negatively regulates canonical NF-κB signaling (50, 52) (Figure 1). NLRX1 associates with TRAF6 and IκB kinase (IKK) through an activation signal-dependent mechanism (50). Following stimulation, NLRX1 is rapidly ubiquitinated and disassociates from TRAF6 to bind the IKK complex and inhibit subsequent canonical NF-κB activation (50). In addition to attenuating NF-κB signaling, NLRX1 also negatively regulates type-I interferon (IFN-I) signaling through inhibiting the interaction between the PRR Rig-I and the mitochondrial anti-viral signaling (MAVS) protein following virus exposure (50–52, 74, 75) (Figure 2). NLRX1 also functions as a positive regulator of autophagy following virus exposure through interacting with the protein TUFM and the mitochondrial immune signaling complex (MISC), which also includes ATG5, ATG12, and ATG16L1 (74, 75) (Figure 2). Interestingly, autophagy also functions as a negative regulator of IFN-I signaling and provides an additional route for the negative regulatory properties of NLRX1. In addition to regulating NF-κB and IFN-I signaling, subsequent studies have also shown that NLRX1 functions as a positive regulator of ROS production in epithelial cells following *Chlamydia trachomatis* infection, likely through interactions with the UQCRC2 protein (76, 77) (Figure 2). Thus, it is clear that NLRX1 regulation is quite complex and appears to occur through cell type, temporal and signal-dependent mechanisms.

NLRC3 is the most recently characterized member of this functional sub-group and has been shown to negatively regulate NF-κB and IFN-I signaling (54, 78). NLRC3 was originally identified as a negative regulator of T cell function, in part through delaying the degradation of IκBα (78). Subsequent studies have since revealed that NLRC3 attenuates TLR signaling through interacting with and modulating TRAF6 activity and inhibiting canonical NF-κB signaling (54). NLRC3 has also been recently shown to fine tune the host innate immune response to intracellular DNA, DNA viruses, and c-di-GMP (53). NLRC3 impedes STING-TANK-binding kinase 1 (TBK1) interactions and inhibits STING trafficking, which results in an attenuation of subsequent downstream activation of IFN-I genes (53).

While NLRP12, NLRX1, and NLRC3 each influence a variety of signaling pathways, the convergence on NF-κB signaling appears to be a common strategy among the NLRs in this functional sub-group to attenuate inflammation (Figure 1). Additional mechanistic studies have revealed prevalent NLR–TRAF interactions in these models and support the emerging hypothesis that these NLRs function to inhibit NF-κB signaling through the formation of a multi-protein “TRAFasome” complex (54). Dysregulated NF-κB signaling and the additional pathways modulated by these NLRs are critical features in cancer initiation and progression. Thus, the NLRs that modulate these signaling cascades are highly relevant to cancer pathobiology and additional mechanistic insight will be critical for developing future therapeutic strategies.

## Negative Regulatory NLRs in Cancer Pathobiology

While several studies have characterized the contribution of the NLRP3, NLRC4, and NLRP6 inflammasomes in tumorigenesis, significantly less is known regarding the role of NLRs that negatively regulate inflammation. Initial studies have focused on NLRP12. In the context of cancer, somatic mutations in human *NLRP12* have been detected in several large scale screening studies evaluating a variety of cancer sub-types, including glioblastoma, breast cancer, lung squamous cell carcinoma, melanoma, prostate adenocarcinoma, and colon adenocarcinoma (http://cancergenome.nih.gov/). However, broader linkage with specific populations, causation, and mechanism for each mutation has not yet been established. In mice, NLRP12 has been shown to attenuate colorectal cancer. Using the AOM/DSS model of CAC, *Nlrp12*^−/−^ mice were shown to develop increased inflammation and tumorigenesis (66, 68). Colon histopathology revealed significant epithelial cell damage and loss of barrier integrity in these animals, which resulted in increased pro-inflammatory cytokine and chemokine production (66, 68). These animals eventually develop extensive pre-cancerous lesions, which result in significantly increased areas of hyperplasia, dysplasia, and adenocarcinoma (66, 68). These studies revealed that NLRP12 attenuates inflammation and tumorigenesis through negatively regulating NF-κB and ERK signaling (66, 68).

While the overall results of each study are quite complementary, it should be noted that a few mechanistic differences were proposed. In one study, the increased tumorigenesis was attributed to an increase in canonical NF-κB signaling (66). NF-κB signaling was evaluated *in vivo* and in macrophages isolated from wild type and *Nlrp12*^−/−^ mice following PAMP stimulation and a significant increase in the levels of p-p105, Rel-A, and p65 activity was observed (66). Furthermore, loss of NLRP12 was shown to significantly increase the transcription of a variety of pro-inflammatory mediators associated with canonical NF-κB signaling and colon tumorigenesis, including *Il-6*, *Tnf-*α, and *Cox2* (66). These findings are consistent with earlier *in vitro* studies, which demonstrated that NLRP12 functions as an antagonist of TLR and TNFR-induced pro-inflammatory signals, in part through inhibiting IRAK-1 hyper-phosphorylation (48). In the second study, NLRP12 was shown to attenuate colon tumorigenesis through negatively regulating non-canonical NF-κB signaling. While some markers of canonical NF-κB signaling were found to be transiently increased in the absence of NLRP12, this study revealed a significant increase in NIK activation and p100 to p52 cleavage in primary cells and in colon tissues isolated from *Nlrp12*^−/−^ mice during disease progression (68). These data are highly consistent with previous *in vitro* studies associating NLRP12 activity with NIK suppression and attenuation of non-canonical NF-κB signaling (49, 79). Loss of NLRP12 resulted in a significant increase in *Cxcl12* and *Cxcl13* expression in the colons from *Nlrp12*^−/−^ mice (68). These chemokines are highly associated with non-canonical NF-κB activation and cancer (49, 68, 80–82). CXCL12 (SDF-1) and CXCL13 (BLC) and their respective receptors CXCR4 and CXCR5 have been implicated in tumor growth, metastasis, and are critical for the regulation of the tumor microenvironment in multiple cancer sub-types as a component of the tumor “Immunome” (3, 83–85). Regulation of the NF-κB signaling pathway is highly complex. The apparent discrepancies between these two studies can be reconciled by previous findings, which show that non-canonical NF-κB signaling can influence both the canonical pathway and MAPK signaling (86, 87). It is also highly likely that NLRP12 regulates canonical and non-canonical NF-κB signaling through currently undefined cell type, temporal and/or stimuli-specific mechanisms.

To date, neither NLRX1 nor NLRC3 have been directly evaluated in the context of cancer. As previously stated, both of these NLRs negatively regulate NF-κB signaling and would be expected to attenuate tumorigenesis through mechanisms similar to those described for NLRP12. However, each also regulates pathways other than NF-κB that could dramatically influence cancer pathobiology. For example, NLRX1 has been shown to additionally regulate ROS production and autophagy. The dysregulation of oxidative stress signaling is a well-established and important element of tumor development (88). Similarly, autophagy is thought to have a dual function in cancer, where it can attenuate tumor initiation by suppressing tissue damage and inflammation signaling or it can function as a tumor promoter to sustain metabolism, growth, and survival through metabolite recycling (89, 90). Thus, it is highly likely that NLRX1 will contribute to tumorigenesis; however, it is difficult to speculate which of its many biologic functions will have a greater influence on disease pathogenesis.

## Conclusion

The recent characterization of this unique sub-group of NLRs that function to attenuate inflammation emphasizes the point that a significant number of the identified NLR proteins in humans have yet to be adequately characterized. Identifying the unique regulatory and signaling pathways modulated by these NLRs is an essential step toward ultimately developing effective therapeutics targeting these proteins and the pathways they modulate. Characterizing unidentified ligands, cell type and temporal regulatory mechanisms, and redundant functions of these NLR family members will significantly improve our understanding of the contribution of these proteins in maintaining immune system homeostasis. It is also clear that NLRs significantly impact cancer pathobiology, beyond colorectal cancer. Additional studies are necessary to better define the contribution of both inflammasome forming NLRs and non-inflammasome forming NLRs in modulating the hallmarks of cancer.

## Conflict of Interest Statement

The author declares that the research was conducted in the absence of any commercial or financial relationships that could be construed as a potential conflict of interest.
